# Pyrodine, a New Antiseptic

**Published:** 1889-01

**Authors:** 


### EDITORIAL.

### *PYRODINE, A NEW ANTIPYRETIC.*

In the *Medical Chronicle*, of November, 1888, Dr. J. Dreschfeld reports some interesting clinical observations on pyrodine [which should be spelled pyrodin]. He has used it in various forms of fever. It was given in 12 cases of croupous pneumonia. It quickly reduced the temperature, and in three cases there was no further rise after the first administration. In the other 9 cases the reduction lasted over 12 hours. The dose varied from 4 grains in children to 12 grains in adults. All the patients bore it well. Profuse sweating accompanied the reduction of temperature. Of the 12 cases 11 recovered. The fatal case was moribund when admitted to the hospital.

The drug was tried in 25 cases of scarlet fever, in which the initial temperature was very high. The effect in these cases was very good. The temperature was quickly reduced, sometimes below normal, without any unpleasant symptom, such as prostration or collapse, being produced. It was not found that cases that took the drug were more liable to suffer from post-scarlatinal nephritis. Recurrence of high temperature was quickly allayed by repetition of the pyrodin.

In 20 cases of typhus fever the results were highly satisfactory. Given in small quantities, one or two doses a day, pyrodin reduced the temperature, and enabled the patient to pass through the fever period with a reduced temperature. It gave the patients great comfort, diminished the delirium, and did not delay the crisis. The patients required but a small amount of stimulants, and their convalescence was very rapid.

In typhoid fever the toxic effects of pyrodin, which were manifested earlier than in any other disease, were an objection to its free administration. It reduced the temperature, but symptoms of aniline poisoning (jaundice, hebetude, occasionally sickness) came on, due to the action of the pyrodin on the blood; these symptoms were by anæmia.

Pyrodin acted well in two cases of migraine in which antipyrin had failed.

The results of Dr. Dreschfeld's investigation in regard to the use of this drug are summarized as follows:

1. It is a powerful antipyretic, reducing fever-temperatures quickly, and maintaining the temperature at a low level for some hours.

2. It is easily taken; but given in often repeated doses at short intervals, it easily shows toxic effects, which depend on its action on the blood, producing hæmoglobinuria. Unless the temperature be very high, it should not be given oftener than once in 18 or 24 hours, and its use should not be continued for more than a few days.

3. It produces marked perspiration, but no nausea, vomiting, nor collapse. The dose for children is from 2 to 4 grains; for adults from 8 to 12 grains.

4. It reduces the pulse as well as the temperature, and often causes diuresis.

5. It is a much more powerful antipyretic than antipyrin, antifebrin, or phenacetin, but it is more toxic than these bodies, and must be given less frequently.

